# Congenital rubella hospitalizations in Brazil: interpreting administrative data within the context of rubella elimination

**DOI:** 10.1017/ash.2026.10361

**Published:** 2026-04-10

**Authors:** Gustavo Yano Callado, Marina Martins Siqueira, Lucas Hernandes Corrêa, Felipe Mendes Delpino, Alexandre R. Marra, Eduardo Félix Martins Santana

**Affiliations:** 1 Faculdade Israelita de Ciências da Saúde Albert Einstein, Hospital Israelita Albert Einstein, São Paulo, Brazil; 2 Centro de Estudos e Promoção de Políticas de Saúde (CEPPS), Sociedade Beneficente Israelita Brasileira Albert Einstein, São Paulo, Brazil; 3 Department of Internal Medicine, University of Iowa Health Care, Iowa City, IA, USA; 4 Department of Obstetrics, Federal University of São Paulo (UNIFESP), São Paulo, Brazil

Dear Editor,

We thank the authors of the correspondence for their careful reading of our article, “*Hospitalizations for congenital infections in Brazil’s Unified Health System: nationwide trends and regional disparities, 2008–2024,*” and for their interest in the topic.^1^ We appreciate the opportunity to clarify the interpretation of our findings regarding congenital rubella-related hospitalizations.

First, we fully agree that the elimination of endemic rubella and congenital rubella syndrome (CRS) in Brazil represents a major public health achievement. Our study does not question this status, nor does it suggest the reestablishment of endemic rubella transmission in the country. Rather, our analysis was based on hospitalization records from the Brazilian Unified Health System Hospital Information System (SIH/SUS), an administrative database that captures diagnoses used for hospital admission and reimbursement purposes. The objective of the study was to describe national trends in hospitalizations recorded within this administrative database over time, not to estimate the incidence of laboratory-confirmed CRS or to assess the epidemiological status of rubella transmission.

It is important to emphasize that SIH/SUS does not measure confirmed epidemiological cases. The database records diagnostic codes assigned during hospitalization, which may reflect clinical suspicion, provisional diagnoses, or coding practices related to reimbursement processes. Consequently, hospitalization records coded as ICD-10 P35.0 do not necessarily correspond to laboratory-confirmed CRS cases as defined in surveillance systems such as SINAN. The distinction between administrative hospital records and surveillance-confirmed cases is well recognized in epidemiological research, and analyses based on SIH and similar administrative databases are widely used to describe healthcare utilization patterns rather than confirmed disease incidence.^2–4^

We also acknowledge that part of the rubella-related hospitalizations observed in SIH/SUS may reflect coding inaccuracies or diagnostic uncertainty. This limitation was explicitly addressed in our manuscript. In the limitations section, we stated that reliance on DATASUS SIH introduces “risks of underreporting, misclassification, and regional variability,” given that the database depends on ICD-10 coding accuracy and administrative reporting processes. Thus, we recognized the possibility of misclassification and discussed it as an inherent characteristic of secondary administrative data sets.

At the same time, the presence of hospitalization records coded as congenital rubella in SIH/SUS cannot be automatically interpreted as erroneous without further investigation. The certification of rubella elimination refers to the interruption of sustained endemic transmission, rather than the absolute absence of suspected or sporadic cases.^5,6^ Administrative records may therefore include hospitalizations coded as congenital rubella based on clinical suspicion or preliminary diagnostic considerations that are later excluded by epidemiological surveillance. For this reason, hospitalization data and surveillance-confirmed CRS data represent different constructs and should not be interpreted as directly equivalent.

Importantly, our discussion explicitly acknowledged the success of Brazil’s vaccination and surveillance programs. We described the nationwide vaccination campaign implemented in 2008 and noted that Brazil was certified as having eliminated rubella after five consecutive years without documented endemic transmission. These statements were included precisely to contextualize the rubella findings and to emphasize that rubella hospitalizations represented a small proportion of admissions and declined substantially over time.

Finally, we believe that the discrepancy between SIH/SUS hospitalization records and surveillance-confirmed CRS cases may itself represent a relevant topic for further investigation. Differences between administrative hospital data and epidemiological surveillance systems are well documented in public health research and can provide valuable insights into diagnostic practices, coding behaviors, or healthcare utilization patterns.

Our study aimed to describe patterns of hospitalizations recorded in a national administrative database and did not attempt to estimate the incidence of confirmed CRS or challenge Brazil’s elimination status. Although Brazil has achieved certification of rubella elimination, this status does not imply that suspected cases can no longer occur. On the contrary, maintaining elimination requires continued vigilance, including the active identification and appropriate notification of suspected cases. Given the substantial public health effort that led to the elimination of rubella through decades of vaccination campaigns, it is essential that any signals suggesting possible cases be carefully examined rather than dismissed outright as erroneous records.

We appreciate the opportunity to clarify these points and agree that continued integration and comparison of administrative and surveillance databases are essential to strengthen the interpretation of congenital infection data in Brazil.

Sincerely,

Gustavo Yano Callado, Marina Martins Siqueira, Lucas Hernandes Corrêa, Felipe Mendes Delpino, Alexandre R. Marra, Eduardo Félix Martins Santana

## References

[1] Callado GY , Siqueira MM , Corrêa LH , Delpino FM , Marra AR , Santana EFM. Hospitalizations for congenital infections in Brazil’s unified health system: nationwide trends and regional disparities, 2008–2024. Antimicrob Steward Healthc Epidemiol 2026;6:e40. 41658320 10.1017/ash.2026.10300 PMC12877911

[2] Portela MC , de Aguiar Pereira CC , Lima SML , de Andrade CLT , Martins M. Patterns of hospital utilization in the unified health system in six Brazilian capitals: comparison between the year before and the first six first months of the COVID-19 pandemic. BMC Health Serv Res 2021;21:976. 34535135 10.1186/s12913-021-07006-x PMC8445784

[3] Pachito DV , Longato M , Cordeiro G , Almeida PHRF , Ferreira RM , Burian APN. Hospitalization due to pneumococcal disease in the unified health system in Brazil: a retrospective analysis of administrative data. Braz J Infect Dis 2025;29:104482. 39608221 10.1016/j.bjid.2024.104482 PMC11638580

[4] Ali MS , Ichihara MY , Lopes LC et al. Administrative data linkage in Brazil: potentials for health technology assessment. Front Pharmacol 2019;10:984. 31607900 10.3389/fphar.2019.00984 PMC6768004

[5] Ministry of Health (Brazil). Brazil receives certificate of elimination of rubella in national territory, 2015. Brasília: Ministry of Health, 2025. website. https://www.gov.br/saude/pt-br/assuntos/noticias/2015/dezembro/brasil-recebe-certificado-de-eliminacao-da-rubeola-em-territorio-nacional.

[6] World Health Organization. Guidance for evaluating progress towards elimination of measles and rubella. Wkly Epidemiol Rec 2018;93:544–552.

